# *Dirofilaria* spp. Detection in Dog Blood Samples from Southern Poland—A Retrospective Data Analysis

**DOI:** 10.3390/idr18030052

**Published:** 2026-05-27

**Authors:** Olga Pawełczyk, Paulina Iwase, Bartosz Wierzba, Jolanta Szłapka-Kosarzewska

**Affiliations:** 1 Department of Microbiology, Faculty of Pharmaceutical Sciences in Sosnowiec, Medical University of Silesia, 40-055 Katowice, Poland; 2 Vetlab Sp. z o. o., Veterinary Diagnostic Laboratory, 40-599 Katowice, Poland; 3 Vetlab Sp. z o. o., Veterinary Diagnostic Laboratory, 52-017 Wroclaw, Poland

**Keywords:** *Dirofilaria* spp., dirofilariasis, vector-borne diseases, mosquito-borne diseases, blood smears, zoonoses

## Abstract

Background: *Dirofilaria* spp. is an etiological agent of dirofilariasis, a mosquito-borne parasitic disease of increasing zoonotic concern in Europe. The aim of this study was to assess the occurrence of *Dirofilaria* spp. in dogs from Southern Poland using retrospective data from a commercial veterinary diagnostic laboratory (Vetlab, Katowice, Poland). Methods: Blood tests from 2060 dogs were analyzed between 1 August 2018 and 31 December 2022. All samples were collected by the clinicians during routine veterinary activity and examined by a specific test—microscopic (blood smear/blood smear and Knott’s test), molecular or both—from the Vetlab laboratory offer (test selected by clinician). Results: Out of all examined dogs, 19 (0.92%) tested positive for *Dirofilaria*. Positive samples originated from the Śląskie (n = 13), Opolskie (n = 3), and Małopolskie (n = 3) voivodeships. Co-infections with *Babesia* spp. and *Anaplasma* spp. were identified in two blood samples. Conclusions: This study demonstrates the presence of *Dirofilaria* spp. in dogs from Southern Poland, a region where data about dirofilariasis cases remain limited. Its overall occurrence was low in comparison to endemic areas in Central Poland. However, the presence of confirmed cases highlights the need for increased veterinary awareness, implementation of preventive measures, and further molecular epidemiological studies to better evaluate the risk of exposure to *Dirofilaria* in this region.

## 1. Introduction

Nematodes of the genus *Dirofilaria* spp. (Spirurida, Onchocercidae) belong to parasites transmitted by mosquitoes from the Culicidae family, which exhibit zoonotic potential and may incidentally affect humans [1]. Their primary hosts are carnivores, including domestic dogs (*Canis lupus familiaris*), as well as gray wolves (*Canis lupus*), red foxes (*Vulpes vulpes*) and racoons (*Procyon lotor*) [2,3]. *Dirofilaria repens* is an etiological agent of subcutaneous dirofilariasis, the disease that causes local inflammation—mainly in subcutaneous or ocular tissues. Dermatological symptoms of this disease in infected dogs are pruritus, papules, crusting, nodules, pyoderma, erythema and dermal swelling, but sometimes it may also present extradermic manifestations, like anorexia, fever, lethargy or ocular conjunctivitis [1,4]. In the case of humans, infective larvae are detected by the host’s immune system leading to destruction of the parasite, but they may also cause a ‘larva migrans syndrome’ with mild and ambiguous symptoms [5,6,7]. *D. repens* infections are increasingly common in the Mediterranean basin and year after year appear in new locations in Central and Eastern Europe [8]. The main reason for the spread of this species to new areas is global warming [9]. This phenomenon allows for the introduction of new mosquito species, vectors of this nematode, into new geographical regions [9]. Moreover, traveling with pets to *Dirofilaria*-endemic areas, as well as dogs’ migration from Ukraine as a result of armed conflict, significantly influences the risk of transmitting this zoonotic nematode to other locations [1,10,11].

In Poland, the first case of canine dirofilariasis caused by *D. repens* was described in 2009 [12]; then, in dogs from 18 districts of the Mazowieckie voivodeship, a 25.8% prevalence of this parasite was observed [13]. In 2014, Demiaszkiewicz et al. (2014) reported the distribution of dirofilariasis throughout the country (11.7% *D. repens*-positive dogs) with the highest prevalence in Lublin (16.2%), Podlasie (12.6%) and Mazovia (4.6%) [14]. Currently, due to high *D. repens* prevalence in dogs, as well as the presence of autochthonous human dirofilariasis cases in these voivodeships, Central Poland has become established as an endemic region for this parasite [15,16,17]. Moreover, sporadic autochthonous cases of heartworm disease caused by *D. immitis* have also been reported in Poland. So far, they were confirmed in 2012 in Pomerania, based on SNAP test (IDEXX) results [18], in 2014 in Silesia [19] and in 2025 in Lublin voivodeship [20], both during necropsy. In 2022, the first molecularly (PCR) confirmed *D. repens* and *D. immitis* co-infection case was reported in a dog that had never traveled abroad [21].

Unfortunately, knowledge about the *D. repens* and *D. immitis* epidemiological situation among dogs in Poland is still limited. Therefore, the main aim of this study was to provide an update on diagnosed *Dirofilaria* spp. cases among dogs from Southern Poland, based on retrospective data from a veterinary diagnostic laboratory in Katowice, Poland.

## 2. Materials and Methods

In this retrospective study, the occurrence of *Dirofilaria* spp. in dogs was estimated using data gathered by Vetlab, a commercial veterinary diagnostic laboratory (Katowice, Poland). There were 2060 dogs analyzed using blood tests between 1 August 2018 and 31 December 2022. All samples were collected by clinicians during routine veterinary activity. The diagnostic method was chosen by clinicians from Vetlab’s laboratory offer, which included: microscopic analysis (blood smear; blood smear and Knott’s test), molecular analysis or both. In general, 2041 blood samples were sent for blood smear; 14 for blood smear and Knott’s test; 1 for blood smear, Knott’s test and PCR; and 4 for molecular (PCR) analysis only.

The samples were sent from veterinary clinics in Southern Poland, including Śląskie, Małopolskie, Opolskie, Podkarpackie, Świętokrzyskie and Lubelskie voivodeships. The laboratory received blood samples taken from cephalic, saphenous or jugular veins by veterinary clinic staff, stored at room temperature (between 15 °C and 25 °C degrees) with ethylenediaminetetraacetic acid (EDTA) for microscopic analysis. The exact time of blood collection is unknown, but all samples were tested on the day of collection. Blood samples of poor quality and samples with insufficient volume were excluded from analysis.

Samples were not collected specifically for this study. Results were collected without owner information or canine patient identification in order to ensure data privacy. Only data about geographic provenance, season of diagnosis and breed were known for the *Dirofilaria*-positive dogs. No information about clinical status, signs and other laboratory findings was available.

### 2.1. Blood Smears

Thin blood smears from the EDTA-treated blood samples and the Wright–Giemsa staining method with methanol, thiazine and eosin dyes (ELITechGroup SAS, Puteaux, France) were prepared. In order to increase the possibility of detecting parasites, two blood smears were conducted. The first smear was made using a standard technique, which was thin, in order that cells do not overlap; while the second slide was thicker for the detection of microfilariae under the light microscope (ZEISS Axiolab 5, Shanghai, China). This way, *Dirofilaria* larvae are better visualized due to their tendency to pack into the thicker areas. After the staining process, smears were examined microscopically: the thick slide was viewed under 10×, while the thinner slide under 40× and 100× magnification. Detection of *Dirofilaria* in a thick smear did not rule out the evaluation of the second slide, due to the possibility of co-infection with other blood. Blood smears were performed on the same day as blood collection.

### 2.2. Knott’s Test

Microfilariae in dog blood samples were also detected microscopically in concentrated blood by using the modified Knott’s test. A quantity of 1ml of EDTA blood samples was mixed carefully with 9 mL of 2% formalin. The next step included 5 min centrifugation at 1500 rpm, which was followed by extraction of the supernatant. Then, 1% of methylene blue was added to the precipitate and mixed. The process was finished by dropping the mixture on a slide and after application of a coverslip. The samples were analyzed microscopically, using 10×, 20× and 40× magnification.

### 2.3. PCR

Data analysis showed that only 5 dog blood samples were ordered by clinicians to be sent for PCR. Molecular analysis was performed in the external laboratory of SYNLAB International GmbH in Germany (Moosacher Str. 88, 80809 München), and the Vetlab laboratory (Katowice, Poland) received information about PCR results.

### 2.4. Statistical Analysis

Statistical analysis was performed in Microsoft Excel 2019. The Clopper–Pearson test was used to calculate prevalence with confidence intervals.

## 3. Results

### 3.1. Overall Data

Between 1 August 2018 and 31 December 2022, 2060 dog blood samples were examined in the Vetlab laboratory, Katowice, Poland. Out of 2060 samples examined for blood parasites, 19 of them were *Dirofilaria*-positive (0.92%). The prevalence showed similar values between 2019 and 2022, which means that the number of positive samples increased with the number of samples tested. A difference in the prevalence value was recorded in 2018, when the number of examined blood samples was significantly smaller (Table 1). Out of 19 positive samples, *Dirofilaria* parasites were detected in 15 blood smears confirmed by Knott’s test, and in five blood samples examined by PCR (including one sample, which was also positive in microscopic analysis) (Table 2).

### 3.2. Microscopic Analyses

**Figure 1:**
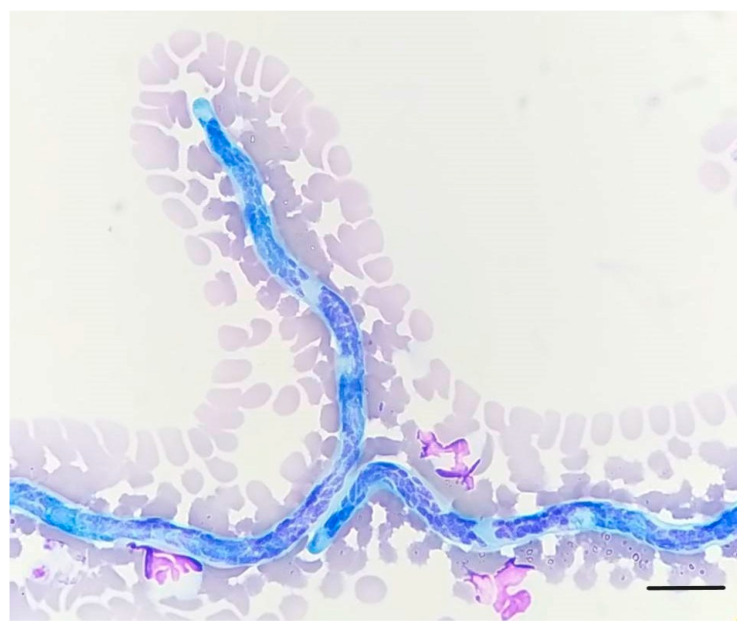
*Dirofilaria* spp. microfilariae in blood smear stained by Wright–Giemsa method (100× magnification; bar = 20 µm).

A total of 15 blood smears and Knott’s tests were *Dirofilaria*-positive. All microscopically analyzed cases belonged to the *Dirofilaria* spp. genus, most likely *Dirofilaria repens* species. The microscopic evaluation of dog blood samples revealed 360 ± 10 μm long and 8 ± 1.5 μm wide microfilariae with typical features for *D. repens*, such as: rounded upper pole with 2–3 front nuclei separated from the main nuclear column and located at a distance of about 3 μm from the anterior cephalic space, and a pointed tail with a single terminal nucleus located at a distance of about 35 ± 5 μm from the end of the nematode body [22,23] (Figure 1). There was no possibility for molecular analysis of examined samples in order to confirm *Dirofilaria* species, because of the retrospective character of this study. Only one molecular confirmation of positive microscopic analysis was ordered by a veterinary clinic from Śląskie voivodeship (Żarki). The PCR confirmed *D. repens* species in the examined sample (Table 2).

### 3.3. Molecular Analysis

Five dog blood samples were ordered for molecular analysis and showed the presence of *Dirofilaria repens* (SYNLAB International GmbH, Moosacher Str. 88, 80809 München, Germany). One of them was also examined by a blood smear and Knott’s test, but the clinician ordered the molecular analysis for confirmation. The PCR confirmed *D. repens* infection (Żarki, Silesia) (Table 2).

### 3.4. Dirofilaria Positive Cases—Location, Examination Date, Dog Breed, Co-Infections

In general, 19 dogs were *Dirofilaria*-positive, including 13 cases from Śląskie (68.4% of all positive samples), three from Opolskie (15.8%) and three from Małopolskie (15.8%) voivodeships. Moreover, 2 of 19 positive samples were co-infected with other pathogens, like *Anaplasma* spp. (n = 1) and *Babesia* spp. (n = 1). A total of 13 out of 19 dirofilariasis cases were reported in pure breed dogs, which accounted for 70% of all *Dirofilaria*-positive dogs (Table 2).

## 4. Discussion

Filariasis is a group of vector-borne diseases that occurs worldwide. The highest prevalence of these parasitic diseases is noted in tropical and subtropical zones. However, numerous reports have pointed to their steady expansion to new areas, which is related to climate change and increased mobility of animals [1,24,25]. In Europe *Dirofilaria repens* is endemic in Mediterranean countries with a very high number of cases in Greece and Italy [26,27]. Nowadays, this parasite species is also frequently reported in countries in Central Europe, like Ukraine, the Czech Republic, Slovakia, Hungary, Germany, as well as Poland [11,17,28,29,30,31,32,33,34].

The main purpose of this study was to assess the number of *Dirofilaria* spp. cases in dogs in Southern Poland based on retrospective data collected from a commercial veterinary diagnostic laboratory. The data came from six voivodeships in Southern Poland, namely Śląskie, Małopolskie, Opolskie, Podkarpackie, Świętokrzyskie and Lubelskie. The study highlights the presence of the *D. repens* nematode in dog blood samples from Śląskie (n = 13), Opolskie (n = 3) and Małopolskie (n = 3) voivodeships. In total, 19 (0.92%) *D. repens*-positive cases were detected. In Poland, the first case of canine dirofilariasis was reported in 2009 [12,35] and since then the prevalence of this disease among dogs has been steadily increasing. In the study of Demiaszkiewicz et al. (2014), *D. repens* microfilariae were detected in 11.7% of blood samples of dogs originating from 16 provinces in Poland. The study confirmed the occurrence of this parasite in all Polish provinces, with the highest prevalence in Mazovia (25.5%) and the lowest in Lesser Poland (1.2%) [14]. Furthermore, the researchers showed a risk of infection for this parasitosis in Podlaskie and Lubelskie voivodeships, where its prevalence was respectively 12% and 15% [13,36]. In comparison to the presented study, 11.7% is a very high result, most likely caused by a very large number of cases from Mazovia. The region is an endemic area for canine dirofilariasis in Poland, where this parasitosis is constantly reported [17,35,36,37]. It should be noted that the study of Demiaszkiewicz et al. (2014) concerned canine dirofilariasis cases detected during clinical examination, while this retrospective study showed the presence of *Dirofilaria* in blood collected from dogs without any information about additional dirofilariasis cases diagnosed in veterinary clinics. Clinical study from the Czech Republic and Slovakia [38], two countries bordering Poland, showed notable differences in the prevalence of *Dirofilaria*-infected dogs. In the Czech Republic, 1.6% of examined dogs were *D. repens*-positive, while in Slovakia 5.9% of examined dogs were infected by this species. Moreover, *D. immitis* was found in both countries, but only the cases found in Slovakia were autochthonous [38]. Considering that during clinical examination it is possible to extend the diagnostics and collect detailed medical history about the animal, then the frequency of *Dirofilaria* detection could be higher. Moreover, there is a group of animal owners who do not consent to pay for blood tests in an external laboratory for financial reasons, which could also affect the results of this retrospective study. Increased awareness of vector-borne diseases, access to broader diagnostic facilities, as well as better information about cases diagnosed microscopically during clinical examination in veterinary clinics, could also help improve detection. There is a high probability that the number of dirofilariasis cases in the studied area is underestimated. Most importantly, in the presented study the vast majority of blood samples were examined microscopically by blood smear. This method has a significantly lower sensitivity in comparison to Knott’s test, a standard concentration method for the detection of microfilariae, or molecular analysis. These diagnostic limitations may lead to underestimation of the true occurrence of *Dirofilaria* cases, because infections with low microfilaremia could remain undetected. In order to estimate the most accurate results of *Dirofilaria* prevalence in the dog population of Southern Poland, a combination of microscopic, serological and molecular screening tests should be conducted, which is planned in future research.

*Dirofilaria repens* infection in dogs may cause dermatological symptoms, like nodules in subcutaneous tissue, multifocal dermatitis, anorexia, lethargy, and multiple organ dysfunction syndrome, but most often it is asymptomatic [4,17,39,40]. In this study, out of 19 *Dirofilaria*-positive dogs, two of them were also co-infected with other vector-borne pathogens, *Babesia* spp. and *Anaplasma* spp. Furthermore, in blood samples that were not molecularly analyzed, we cannot rule out the mixed infection *D. repens* with *D. immitis*. The first case of such co-infection was recently reported in Warsaw (Mazowieckie voivodeship) [21]. In addition, in Slovakia, mixed infections of *D. repens* with *D. immitis* accounted for as much as 31.33% of all reported dirofilariasis cases between 2019 and 2022 [34]. There is no information about clinical conditions of the examined dog population; however, symptomatic dirofilariasis cases are often diagnosed during *B. canis* co-infection. Most probably it could be the result of heavy microfilaremia in capillary vessels and red blood cell adhesion during babesiosis [17]. The last case study by Gałęcka and Platt-Samoraj (2025) confirmed the presence of symptomatic dirofilariasis in a dog with triple infection, *D. repens*, *Babesia* spp. and *A. phagocytophilum,* in North-Eastern Poland [41].

The highest value of microfilaremia caused by *D. repens* in blood should be observed at night or early in the morning, when mosquitoes are most active. This hypothesis was confirmed in the study conducted by Kludkowska et al. (2018) [23], where the highest values of larvae were detected in material collected at 7 a.m. (10–12 microfilariae), and at 10 a.m it was lower by almost half [9,10,17]. The microfilariae were no longer present in blood collected at 1 p.m. In the presented study, the exact time of blood collection is unknown because of its retrospective character. It can be assumed that most samples were collected during veterinary clinics’ opening hours between 8 a.m. and 8 p.m. Therefore, a low occurrence of *D. repens* in blood samples may be partly a result of the sampling collection time.

Besides pets, wild animals are also reservoirs for the *D. repens* nematode. So far, this parasite was identified in wild carnivores from 7 out of 14 examined voivodeships in Poland. The highest prevalence of *D. repens* was observed in Mazowieckie (8.9%) and Łódzkie (7.3%), only two positive samples were noted in red foxes from Małopolskie (n = 1) and Lubelskie (n = 1), whereas no positive sample was observed in Śląskie, Opolskie, Podkarpackie and Dolnośląskie voivodeships [3]. The reservoir role of wild animals for *D. repens* nematodes increases the risk of their circulation in the natural habitat, mainly among their hematophagous vectors. It could be associated with microfilariae transmission to potential hosts, such as domestic animals, as well as humans. Considering the prevalence of *D. repens* among wild animals in Southern Poland, the risk of infection appears to be low. On the other hand, the migration of wild animals from different regions in Poland, as well as neighboring countries, where the prevalence of this parasite species is higher, increases the risk of potential carriers in the environment [3,42].

In Poland, the first cases of subcutaneous dirofilariasis in humans were recorded in 2007 [43,44], and since then dozens of cases have been recorded [19,45,46]. Regardless of whether the *D. repens* infection was autochthonous or imported from endemic areas, by far the highest number of human cases of this disease was recorded in Mazovia [47]. Due to the long-term information gap about the occurrence of *Dirofilaria* spp. in Southern Poland, the epidemiological screening of this pathogen in dog blood samples seems to be reasonable. It should be emphasized that close contact between humans and dogs that may be asymptomatic *D. repens* carriers poses a high risk of zoonotic potential [1]. Moreover, due to significant population and animal movement, as well as the region’s proximity to country borders, such as Slovakia and Ukraine, where this parasite is reported more often in the population of domestic and wild animals (Canidae), it should be updated.

## 5. Conclusions

The obtained results showed a low risk of infection with the *Dirofilaria* spp. parasite in dogs from Southern Poland. On the other hand, dirofilariasis should be taken into consideration during the differential diagnosis at veterinary clinics in non-endemic regions, where prophylaxis is not implemented, because of limited knowledge about mosquito-borne diseases [48]. Moreover, we should consider this parasite species during differential diagnosis in humans, due to its zoonotic potential. It should be emphasized that this is only a preliminary pilot study regarding the epidemiology of dirofilariasis in Southern Poland and further research, including microscopic, serological and molecular analyses of potential *Dirofilaria* spp. carriers, is required.

## Figures and Tables

**Table 1:** Number of *Dirofilaria*-positive dog blood samples examined between 2018 and 2022 in veterinary diagnostic laboratory.

Year	Number of Examined Blood Samples [n]	Number of *Dirofilaria*-Positive Samples [n]	Percent of *Dirofilaria* Positive Samples [%]	95% Confidence Interval [%]
2018	36	1	2.78	(0.0007, 0.1450)
2019	205	2	0.98	(0.0012, 0.0353)
2020	438	4	0.91	(0.0025, 0.0233)
2021	607	5	0.82	(0.0027, 0.0191)
2022	774	7	0.90	(0.0036, 0.0186)
Total	2060	19	0.92	(0.0056, 0.0143)

**Table 2:** *Dirofilaria* spp.-positive cases in dogs from Southern Poland examined between 2018 and 2022.

Year	Month	Breed	Location (Voivodeship)	Co-Infections	Detection Method
2018	September	German Shepherd	Bielsko-Biała (Silesia)	-	Blood smear Knott’s test
2019	May	Mixed	Strzelce Opolskie (Opole)	-	Blood smear Knott’s test
	August	Mixed	Cieszyn (Silesia)	-	Blood smear Knott’s test
2020	May	German Shepherd	Opole (Opole)	-	Blood smear Knott’s test
	September	Akita Inu	Blachownia (Silesia)	+(*Babesia* spp.)	Blood smear Knott’s test
	September	Golden Retriever	Bytom (Silesia)	-	Blood smear Knott’s test
	October	Mixed	Sosnowiec (Silesia)	-	PCR
2021	March	Newfoundland	Katowice (Silesia)	-	Blood smear Knott’s test
	August	Shetland Sheepdog	Żarki (Silesia)	-	Blood smear Knott’s test PCR
	September	Mixed	Radzionków (Silesia)	-	Blood smear Knott’s test
	December	German Shepherd	Chorzów (Silesia)	-	PCR
	December	Belgian Shepherd	Kraków (Lesser Poland)	-	Blood smear Knott’s test
2022	March	Shetland Sheepdog	Czernichów (Lesser Poland)	-	Blood smear Knott’s test
	April	Mixed	Knurów (Silesia)	-	PCR
	May	Siberian Husky	Radzionków (Silesia)	+(*Anaplasma* spp.)	Blood smear Knott’s test
	July	Siberian Husky	Reńska Wieś (Opole)	-	Blood smear Knott’s test
	July	Labrador Retriever	Bytom (Silesia)	-	PCR
	August	German Shepherd	Pawłowice (Silesia)	-	Blood smear Knott’s test
	November	Mixed	Trzebinia (Lesser Poland)	-	Blood smear Knott’s test

## Data Availability

The original contributions presented in this study are included in the article/Supplementary Material. Further inquiries can be directed to the corresponding author(s).

## Supplementary Materials

The following supporting information can be downloaded at: https://www.mdpi.com/article/10.3390/idr18030052/s1, Figure S1. Distribution of veterinary clinics with *Dirofilaria* spp.-positive reports confirmed by the Vetlab laboratory [My Maps, Google Maps 2024, Windows 11 Pro].
