# Supplementary material for: Dirofilaria spp. Detection in Dog Blood Samples from Southern Poland—A Retrospective Data Analysis
Source: Infect Dis Rep. 2026 May 27;18(3):52. doi: 10.3390/idr18030052 (PMC13299744; doi:10.3390/idr18030052)

Figure S1. Distribution of veterinary clinics with *Dirofilaria* spp. positive reports confirmed by the Vetlab laboratory [My Maps, Google Maps].

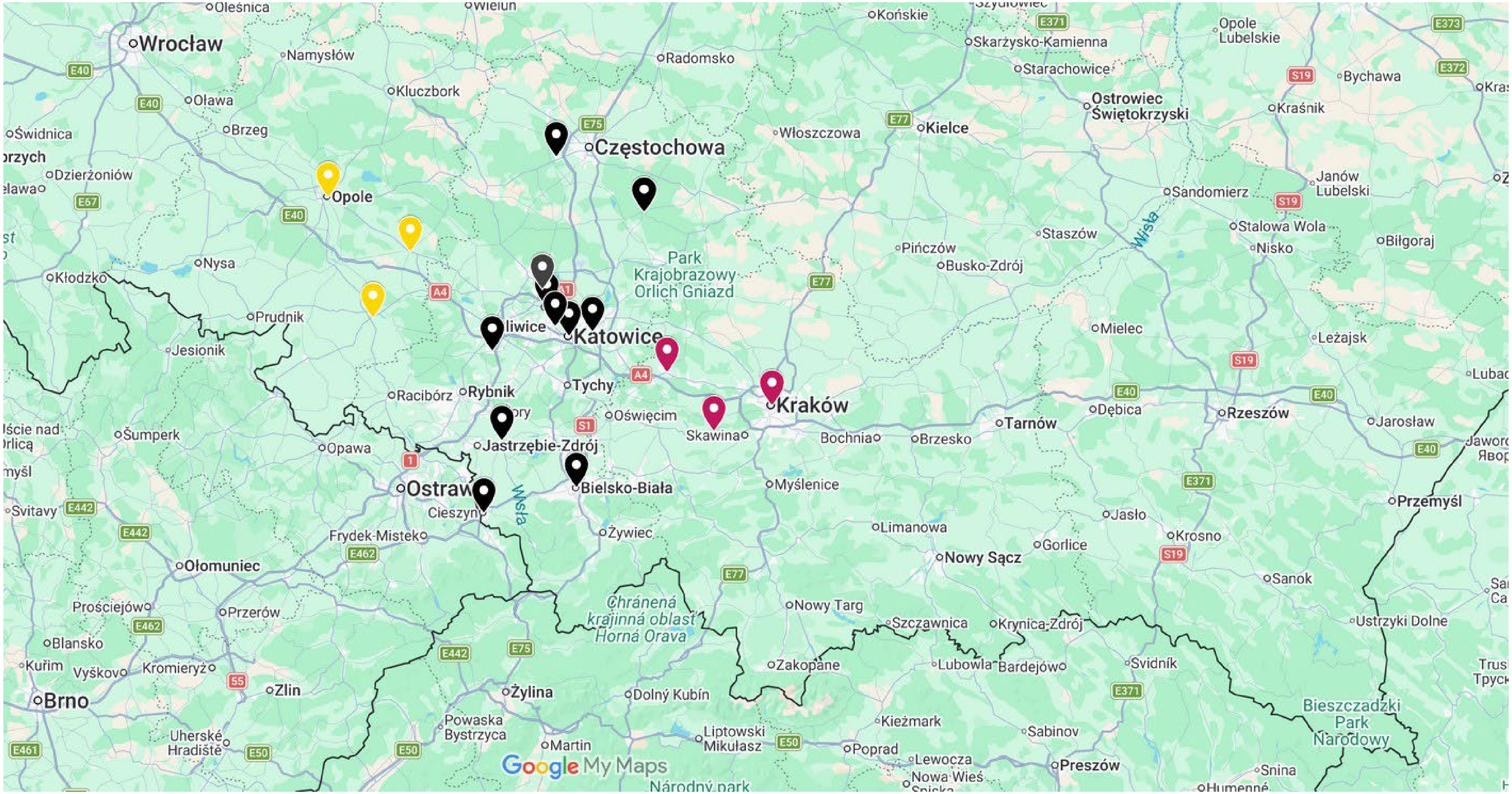

Supplement: Supplementary file 1 [file idr-18-00052-s001.zip › idr-4246270-supplementary.pdf]
